# Epidemiological Trends and Clinical Characteristics of Childhood Leukemia in Saudi Arabia: A Review

**DOI:** 10.7759/cureus.28178

**Published:** 2022-08-19

**Authors:** Nadyah A Owaidhah, Zakaria Y Khawaji, Mohammed A Alahmadi, Ahmad S Badawi, Ghazi H Mogharbel, Osama N Makhdoom

**Affiliations:** 1 Family and Community Medicine, Taibah University, Medina, SAU; 2 Medical School, Taibah University, Medina, SAU

**Keywords:** saudi arabia, hematological malignancies, pediatric leukemia, characteristics of leukemia, epidemiology of leukemia

## Abstract

Leukemia is the most prevalent type of cancer among children in Saudi Arabia. It has variable clinical presentations and accounts for a large scale of mortality and morbidity. Acute lymphoblastic leukemia (ALL) constituted the majority of pediatric leukemic cases with male gender predisposition. The most common first presentation that patients come with are manifestations of anemia, thrombocytopenia, and fever. Bone pain, fatigue, weight loss, organomegaly, and pale skin are among the commonest manifestations of pediatric leukemia. Childhood ALL and acute myeloid leukemia (AML) clinical manifestations seem to be very similar, even though there’re some considerable differences in how common the clinical characteristics are. Chromosomal abnormalities are taken into consideration to determine survival and treatment. PubMed and Google Scholar were searched for the childhood leukemia population in Saudi Arabia. Our review article aims at providing comprehensible and updated statistical data on the different types of leukemia and their clinical presentations in Saudi Arabia.

## Introduction and background

Leukemia is the most common malignancy in childhood. It is characterized by the uncontrollable proliferation of leukocytes or their precursors and is mainly caused by bone marrow dysregulation [1]. Childhood leukemia is categorized based on clinical presentation and pathological findings into acute lymphoblastic leukemia (ALL) which involves a malignant proliferation of B and T lymphocyte precursors and acute myeloid leukemia (AML) which is the form of leukemia that is characterized by infiltration of lymphatic tissues by malignant hematopoietic cells [2,3]. Chronic myeloid leukemia (CML) is a malignant clonal expansion of hematopoietic stem cells that results in an increase in both myeloid and erythroid cells and platelets leading to bone marrow hyperplasia [4]. 

The diagnosis of leukemia consists of a wide range of procedures including complete blood count (CBC), coagulation studies, and chemistry profile (mainly liver and kidney function tests). The morphological appearance of bone marrow aspiration is used to discriminate between various types of leukemia [5]. The management of leukemia depends on the type and its severity. Primarily, multi-agent chemotherapy, immunotherapy, targeted therapy, and bone marrow transplantation are the standards of the childhood leukemia approach [6]. 

GLOBOCAN, WHO's global cancer observatory database, estimates the incidence of leukemia in 2020 to be around 475,000 cases, making leukemia the 13th-most diagnosed malignancy worldwide showing an increasing trend with 311,594 cancer deaths [7]. ALL affects children the most, accounting for 80% of cases, compared to 20% of cases in adults. AML is the most common type of acute adult leukemia. Chronic lymphoblastic leukemia (CLL) was observed to be more common in older patients, particularly those between the ages of 60 and 70 [8]. During 1999-2013 in Saudi Arabia, a number of 8712 cases of leukemia were identified. One-third of the overall number was in the central region in Saudi Arabia. B-cell lymphoblastic leukemia was found to be the most common type [9]. 

Our aim is to provide an updated review of the major epidemiological features of childhood leukemia in Saudi Arabia covering the most important types of leukemia as well as their main clinical features. As regards the methodology, we searched for articles in PubMed and Google Scholar. Research that was outdated and had recent updates on the same issue in literature were excluded. Articles that met the inclusion criteria were included in this research.

## Review

Prevalence of childhood leukemia in Saudi Arabia

According to cancer incidence for children (0 - 14 years) obtained from Saudi Arabia National Cancer Registry (SA-NCR) between 2005 and 2009, the percentage of leukemia according to the international childhood cancer classification (ICCC) was found to be 35.26% of total children with cancers in the same period, thus proving that it is the most common cancer in the country compared to other childhood cancers. It was found that out of 1370 cases, acute lymphoid leukemia and acute myeloid leukemia were the most common types respectively; additionally, males (55.35%) were more affected than females (44.65%). It mostly affects children between the age of one to five years (46.71%); the second-most affected age group were children between 5-10 years (28.54%) followed by the age group between 10-15 years (19.56%) and the least affected age group were children <1 year of age (5.18%) [10]. 

Another study that looked at cases reported between 1999 and 2013 in Saudi Arabia discovered that precursor B-cell acute lymphoblastic leukemia, a type of ALL, has the highest incident rate among children (under 14 years of age), with a percentage of 36.6%. With a prevalence of 31.4%, precursor cell lymphoblastic leukemia was the second most prevalent type of childhood leukemia. The least frequent forms of childhood leukemia, however, were B-cell chronic lymphocytic leukemia and chronic myelogenous leukemia. In 2013, there were 3.6/100,000 cases of female childhood leukemia and 4.2/100,000 cases of male childhood leukemia overall [9]. The regional distribution of the most common childhood malignancies in Saudi Arabia was recorded during a 10-year period (from 1999 to 2008). The data were obtained from Saud Cancer Registry (SCR) which is a national cancer registry in Saudi Arabia. The number of overall childhood cancer cases was 5932 which accounted for 8% of the total cases. Leukemia was noted to be the most common prevalent cancer among Saudi children with rates of 34.1%. Other encountered cancers that were found to be common in the pediatric population in Saudi Arabia were lymphoma (15.2%), brain cancer (12.4%), and kidney cancers (5.3%). The review of the regional distribution of childhood leukemia in Saudi Arabia revealed that the region with the highest number of leukemia cases was Riyadh (28.6%) followed by Makkah (20.1%) and the Eastern region (15.9%). This data could be explained by the presence of comprehensive cancer centers in these regions and cases referred from other regions for management and periodic follow-up [11]. The rest of the data from the provinces in the study are shown in Table 1.

**Table 1:** Regional Distribution of Childhood Leukemia in Saudi Arabia From the Saudi Cancer Registry (SCR) From 1999 to 2008. The information used in this table was obtained from Al-Mutlaq et al [11]. ^1 ^N was calculated based on percentage %.

Regional Distribution of Childhood Leukemia in Saudi Arabia Obtained From SCR (1999-2008)	
Regions	N^1 ^= 2021 (%)
Riyadh	578 (28.6)
Makkah	406 (20.1)
Eastern region	321 (15.9)
Asir	150 (7.4)
Madinah	142 (7.0)
Qassim	97 (4.8)
Jazan	71 (3.5)
Tabuk	69 (3.4)
Hail	45 (2.2)
Jouf	38 (1.9)
Baha	37 (1.8)
Najran	37 (1.8)
North region	30 (1.5)

Regarding ALL, a recent study was conducted to evaluate the incidence and incident rate of ALL among children (14 years of age or younger) from 2001 to 2014 by collecting data from SCR reports. It has been found that the overall incidence of childhood ALL in Saudi Arabia has increased from 1.58 per 100,000 population in 2001 to 2.35 per 100,000 population in 2014. Also, emerging from the available data, the study noted that the incidence of ALL in male children is more common than in females globally including in Saudi Arabia. In further incidence trends details, the incidence of Saudi males increased from 1.88 per 100,000 population to 2.71 per 100,000 in 2001 and 2014 respectively. On other hand, the incidence of ALL in Saudi females was 1.21 per 100,000 in 2001 and rose to 1.81 per 100,000 in 2014. Finally, according to these incidence rates, the study anticipated that in 2025 the incidence of ALL in Saudi males will increase up to 3.8 per 100,000 population and 2.9 per 100,000 in Saudi females. The overall incidence of the same year was predicted to be 3.44 per 100,000 population [12]. 

The survival rate of childhood leukemia in Saudi Arabia

Within the five-year period (2005 to 2009), the overall survival rate of children patients with various types of cancer treated at King Fahad National Center for Children's Cancer (KNFCCC) was determined within a median duration of follow-up of 7.52 years; 504 pediatric patients with leukemia were determined and an overall survival rate within five years period was recorded. The outcome of the treated group was observed with a percentage of 75.7% (± 2.0%), the total incidence of death among the same pediatric leukemic patients were 121 for the same five-year period. The lymphoid leukemia group has a better overall survival rate which was 82.2% (± 2.1%), compared to the acute myeloid leukemia group whose overall survival rate was 51.2% (± 5.5) [11].

Princess Noorah Oncology Center (PNOC) has reported the five-year overall survival data for pediatric ALL admitted in the specific periods: 1989-2000 (period 1), 2001-2007 (Period 2), and 2008-2014 (period 3). A total of 686 children with various types of ALL have been recorded in the study and their treatment outcomes were analyzed. The number of patients classified in periods was as follows: 221 in period 1, 224 in period 2, and 241 in period 3. The five-year overall survival rate has improved from 74% in period 1 to 89.5% in period 3. Moreover, the five-year cumulative incidence of death (whether as a result of toxic death or disease after treatment initiation) has been dramatically reduced, falling from approximately 26% in period 1 to approximately 10.5% in period 3. The significant facilitated improvements in B-cell ALL pediatric outcomes over time were mainly attributed to therapeutic and service measures. Ensuring easier access to medical care, standardization of treatment protocols, environmental control measures, and standard febrile neutropenia protocol, utilizing proper flowmetry techniques to successfully monitor minimal residual disease (MRD) and advances in intensive supportive care were among the implementations employed judiciously to minimize the incidence of leukemia death among children [13]. 

Clinical manifestation of childhood leukemia

Broadly speaking, the first manifestations of acute lymphoblastic leukemia that patients commonly present with are anemia and bleeding diathesis. Also, leukemia has been shown to cause a febrile illness in more than half of patients and increase the bleeding tendency of some patients causing bleeding disorders. Organomegaly may be the first clinical presentation of a patient and that includes both splenomegaly and hepatomegaly. Infectious diseases and intracranial hemorrhage associated with leukemia are the common cause of mortality among leukemic patients [14]. 

In addition to the symptoms listed above, orbital, and ocular lesions are common manifestations in leukemia patients [15]. The aggregation of circulating leukemic cells in the uvea, retinal nerve fibers, optic disc, and other intraocular tissues and fluids can produce ophthalmic symptoms of leukemia [16]. Retinal venous congestion, optic nerve infiltration, retinal pigment epithelial detachment, non-hematogenous retinal detachment, localized or widespread choroidal infiltration, neoplastic hypopyon, iris infiltration, retinal hemorrhages, and white-centered retinal hemorrhages are all examples [16,17]. 

Lymphocytic Leukemia

Globally, in terms of laboratory findings, anemia, thrombocytopenia, and neutropenia are all common. Pallor and weariness, petechiae or purpura, and infections are among them. Furthermore, more than 40% of patients have lymphadenopathy, hepatomegaly, or splenomegaly. Leukemic involvement of the periosteum of bones or joints can cause bone or joint pain and discomfort [18].

Clinical characteristics of childhood leukemia were carried out for 594 Saudi children who were first diagnosed with ALL from January 2004 to December 2008 at King Faisal Specialist Hospital and Research Center, Riyadh (KFSHRC-R). The WBC counts were lower than normal (≤ 50 x 10^9/L ) for the majority of patients (78.3%). However, 11.5% of patients came with hyperleukocytosis with elevated WBCs count (≥ 100 x 10^9/L). Central nervous system (CNS) involvement was investigated by testing cerebrospinal fluid (CSF) for detectable WBC blast cells. Around 30 patients (5.2%) were diagnosed with CNS disease caused by leukemia as they show positive blasts with higher than 5 WBC\ul detectable in CSF. Another 68 patients showed positive blasts with less than 5 WBC\ul in CSF, indicating the development of CNS disease. However, the majority of patients (83%) showed negative leukemic blasts in their CSF which means no involvement of CNS [19]. 

In another retrospective study performed in Jeddah, 130 patients with ALL were admitted to the department of hematology in King Abdulaziz University Hospital (KAUH) from January 2009 to January 2019. The study ruled out patients with CNS involvement or Down syndrome patients. This study included 101 children (between two to less than 14 years). The researchers observed that the most prevalent symptom was fever (33.84%), followed by fatigue (10.76%), bone pain (9.23%), pale skin (8.46%), and weight loss (6.92%) and loss of appetite (6.92%). Although the symptoms recorded weren’t specified based on age group, children comprised a large number of the patients, thus proving these symptoms as the most common clinical manifestations in childhood ALL [20].

**Table 2:** Clinical Picture of Patients With ALL at the Time of Diagnosis in Cases Recorded by King Abdulaziz University Hospital (KAUH) From 2009 to 2019. The information used in this table was obtained from Qari et al [20]. ^1^ Children comprised the majority of patients. ALL: acute lymphoblastic leukemia

Clinical Manifestations of Childhood ALL in KAUH at Time of Diagnosis Among 130 Patients^1 ^(2009 to 2019)	
	N = 130 (%)
Fever	44 (33.8)
Fatigue	14 (10.7)
Bone pain	12 (9.23)
Pale skin	11 (8.4)
Weight loss	9 (6.9)
Loss of appetite	9 (6.9)
Night sweet	5 (3.84)
Headache	3 (2.3)
Vomiting	2 (1.5)

In an attempt to determine the genetic anomalies among children with ALL in KSA, a retrospective study in 2019 analyzed the clinical characteristics among 213 pediatric ALL patients who were treated at King Faisal Specialist Hospital and Research Center (KFSP-RC) in Jeddah during the period of January 2002 to December 2015. The authors of the study aimed to investigate the clinical and genetic abnormalities in childhood ALL and their roles as predictor factors for prognosis and treatment outcomes; 189 patients (88.7%) were found to have pre-B-cell immune phenotype and 24 (11.3%) had a T-cell phenotype. Bone marrow samples were detected only from pre-B-cell phenotypes and were evaluated for cytogenetics and molecular genetics traits. The study showed that 39 patients had triple trisomy (4, 10, and 17), 31 patients had t(12;21) translocation, and five patients had mixed-lineage leukemia (MLL) gene rearrangement. Hyperdiploidy is another prognostic tool used to assess treatment outcomes was evaluated by karyotyping or cellular DNA index. Of the cohort of 133 patients, 28 patients (21%) were found to have hyperdiploidy. As anticipated, the study demonstrated better outcomes with less mortality in patients who were found to have hyperdiploidy, t(12;21) translocation, or trisomies. In contrast to MLL gene rearrangement which was associated with poor prognosis. This study highlighted the importance of cytogenic and molecular assessment to evaluate the prognosis and improve outcomes for the pediatric population with ALL in KSA [21]. 

**Table 3:** Chromosomal and Genetic Abnormalities Among ALL Pediatric Patients From King Faisal Specialist Hospital and Research Center (KFSP-RC) in Cases Registered Between 2002 and 2015 The information used in this table was obtained from Ahmed et al [21]. ALL: acute lymphoblastic leukemia

Genetic anomalies among ALL pediatric patients from KFSP-RC from 2002 to 2015.	
Feature	N = 213 (%)
Immunophenotype	
Pre-B cell	189 (88.7)
T-cell	24 (11.3)
Chromosomal translocation	
Bone marrow trisomy (4, 10, 17)	39 (18.3)
t(12;21)	31 (14.6)
MLL rearrangement	5 (2.3)
Others	69 (32.4)
Ploidy status	
Hyperdiploidy (≥ 1.16 DNA index, chromosome number > 50 or both)	28 (21%, n=133)

The correlation between parenteral consanguinity and germline predisposition mutations associated with childhood ALL in Saudi Arabia was investigated in 2020 There were 60 pediatric patients enrolled in the study who underwent whole-exome sequencing germline DNA. Pathogenic/likely pathogenic (P/LP) mutations were identified, including mutations in TP53, PMS2, and AK2, all of which were found to increase the susceptibility to developing childhood ALL. However, P/LP mutations were present only in 8.3% of the population of the study. Despite the fact that the Saudi Arabia community is considered to be highly consanguineous, no significant correlation was observed between the frequency of P/LP mutations among pediatrics with consanguineous parents compared to those who are not. In addition, the study proposed that homozygous germline mutations in cancer predisposition genes are unlikely to play a substantial role in the risk of childhood leukemia [22]. 

Myeloid Leukemia

Worldwide in myeloid and monocytic/monoblastic leukemia, oral symptoms are common in children. Oral signs can also occur in patients with chronic leukemia. However, they are not quite the same as those seen in acute leukemia. Spontaneous frequent bleeding, mucosal ulceration, gingival enlargement, mucosal pallor, enamel discoloration, temporomandibular joint arthritis, and osteolytic lesions in the mandible are some of the oral symptoms of myeloid leukemia patients. Some clinical manifestations are more common than others, such as petechiae or spontaneous bleeding, mucosal ulceration, and gingival enlargement with or without necrosis. The most common early diagnostic symptoms of leukemia are these symptoms [23]. 

Gingival enlargement, while being infrequently described in the literature, maybe the initial sign of acute leukemia, particularly in AML [23-27]. The presence of an increased number of cells and a buildup of connective tissue characterizes gingiva overgrowth [24]. As previously stated, gingival infiltration is more common in myelomonocytic and monocytic leukemia, and the proposed hypothesis for gingival involvement is based on the microanatomy of the gingiva and the expression of endothelial adhesion molecules, which allows leukocyte infiltration and leads to a soft overgrowth [24,28,29]. As a result of the infiltration, gingival thickness increases, and pseudo-pockets develop, leading to subsequent inflammatory infiltration [27,28]. Granulocytic sarcoma is a consequence of AML that manifests as a tumor of immature hematopoietic precursor cells in extramedullary tissues. In a case report, a six-year-old girl presented with fast progressing bilateral proptosis and downward and outward displacement of the right eye without any inflammatory symptoms. Later, the case was confirmed to be AML after hematological tests [30].

In respect of clinical manifestations of AML, 30 pediatric patients were enrolled in King Fahd Specialist Hospital (KFSH) in Dammam and they represent the total pediatric AML patients referred to KFSH between May 2008 to September 2012. The vast majority of patients (86.7%) complained of bone pains, 70% of patients presented with fever, and 43.3% had a history of increased bleeding tendency. Other manifestations include poor appetite, fatigue, and weight loss as classic presentations of pediatric leukemia. Regarding the physical examination, 66.7% of patients appeared to have pallor skin, 60% and 50% had organomegaly and lymphadenopathy respectively and 23.3% had gum hypertrophy. CNS involvement was observed in 13.3% of pediatric leukemic patients in the study [31].

**Table 4:** Common Clinical Manifestations of Total Pediatric AML Patients Admitted at King Fahd Specialist Hospital (KFSH) From 2008 to 2012 The information used in this table was obtained from Al Daama [31]. AML: acute myeloid leukemia; CNS: central nervous system

Clinical Manifestations of Childhood AML in KFSH (2008 to 2012)	
	N = 30 (%)
Symptoms	
Bone Pain	26 (86.7)
Fever	21 (70)
History of bleeding	13 (43.3)
Poor appetite	11 (36.7)
Fatigue	10 (33.3)
Weight loss	4 (13.3)
CNS manifestations	4 (13.3)
Examination Findings	
Pallor	20 (66.7)
Organomegaly	18 (60)
Lymphadenopathy	15 (50)
Gum hypertrophy	7 (23.3)
Chloroma	2 (6.7)

In 2016, a multicancer childhood de novo AML group study was carried out by the Saudi Arabian Pediatric Hematology Society (SAPHOS) in order to identify the clinical characteristics of childhood AML. In the study, 193 children patients who were diagnosed with AML from January 2005 to December 2012 were determined. The WBC counts of approximately two-thirds of patients were lower than normal ). The CNS involvement among those pediatric patients was equal (17.7%). As a satisfactory result, 85.7% of patients showed remission after the second induction of treatment with less than 5% blasts. The analysis of French-American-British (FAB) classifications of Saudi pediatric AML revealed the predominance of the M2 subtype followed by M5. The majority of children with AML (78.3%) were found to have abnormal karyotyping. The most observed abnormality was t(8;21) (17.7%), other abnormalities include 11q3 rearrangement (17.1%), inv(16) (8.6%), monosomy 7 (4.6%), and the least observed aberration was 3q abnormalities (1.1%). These results may differ from other studies reported in developed countries, yet this variation in germline mutations among pediatric AML was consistent with the observation that different geographic or ethnic backgrounds may hold different genetic mutations [32].

**Table 5:** Frequency of Laboratory and Karyotyping Findings Among de Novo AML Pediatric Patients From Saudi Arabian Pediatric Hematology Society (SAPHOS) During a Period From 2005 to 2012 The information used in this table was obtained from Jastaniah et al [32]. WBC: white blood cell, CNS: central nervous system, FAB: French-American-British classification.

Laboratory and Karyotyping Findings in Pediatric de Novo AML Patients From SAPHOS (2005-2012).	
Feature	N = 175 (%)
WBC count (x10^9/L)	
< 50	118 (67.4)
≥ 50	59 (29.1)
Missing	6 (3.4)
CNS involvement	
Positive	31 (17.7)
Negative	141 (80.6)
Unknown	3 (1.7)
Remission (< 5 blasts after the second induction)	150 (85.7)
FAB subtypes	
M2	48 (27.4)
M5	39 (22.3)
Karyotyping abnormalities	137 (78.3)
t(8;21)	31 (17.7)
11q3 rearrangement	30 (17.1)
Inv(16)	15 (8.6)
Monosomy 7	8 (4.6)
3q abnormalities	2 (1.1)

Future direction

Since there is a significant lack of properly written and updated research regarding the epidemiology of leukemia in Saudi Arabia, there is a need to further look into the epidemiology of this condition to enhance the coverage of other neglected leukemia-related issues in Saudi Arabia. 

## Conclusions

Leukemia is determined to be the most common type of cancer in children in Saudi Arabia, with a high male-to-female ratio. Prevalence of leukemia amongst children age groups is variable, with children between the age of one and five years having the highest rate. Moreover, survival amongst children with lymphoid leukemia is the most common. Bleeding, ulcerations, gingival enlargement, and lymphadenopathy occur commonly between the different types of leukemia with some differences in other symptoms.
